# Efficacy and safety of long-term use of a positive expiratory pressure device in chronic obstructive pulmonary disease patients, a randomized controlled trial

**DOI:** 10.1186/s12890-023-02319-5

**Published:** 2023-01-16

**Authors:** Zhaoning Xu, Zhuo Han, Dedong Ma

**Affiliations:** 1 School of Nursing and Rehabilitation, Shandong University, Jinan, 250012 Shandong China; 2 Cheeloo College of Medicine, Shandong University, Jinan, 250012 Shandong China; 3 Department of Pulmonary and Critical Care Medicine, Qilu Hospital, Shandong University, Jinan, 250012 Shandong China

**Keywords:** Chronic obstructive pulmonary disease, Positive-pressure respiration, Dyspnea, Exercise capacity, 6-min walk test

## Abstract

**Background:**

Exercise intolerance is among the most common symptoms experienced by patients with chronic obstructive pulmonary disease (COPD), which is associated with lung dynamic hyperinflation (DH). There was evidence that positive expiratory pressure (PEP), which could be offered by less costly devices, could reduce DH. The purpose of this study was to evaluate the efficacy and safety of long-term domiciliary use of PEP device in subjects with COPD.

**Methods:**

A randomized controlled trial was conducted and 25 Pre-COPD or mild-to-very severe subjects with COPD were randomized to intervention group (PEP device, PEP = 5 cmH_2_O, n = 13) and control group (Sham-PEP device, PEP = 0 cmH_2_O, n = 12). PEP device was a spring-loaded resistor face mask. Subjects were treated 4 h per day for a total of 2 months. Six-minute walk test (6MWT), pulmonary function, the Modified British Medical Research Council score, and partial pressure of end-tidal carbon dioxide were evaluated at baseline and after two months.

**Results:**

The 6MWD (− 71.67 ± 8.70 m, *P* < 0.001), end-dyspnea (*P* = 0.002), and end-fatigue (*P* = 0.022) improved significantly in the intervention group when compared with the control group. All subjects in the intervention group reported that 4 h of daily use of the PEP device was well tolerated and accepted and there were no adverse events.

**Conclusion:**

Regular daily use of PEP device is safe and may improve exercise capacity in subjects with COPD or pre-COPD. PEP device could be used as an add-on to pulmonary rehabilitation programs due to its efficacy, safety, and low cost.

*Trial registration*: The study was prospectively registered on ClinicalTrials.gov (NCT04742114).

## Background

Chronic obstructive pulmonary disease (COPD) is characterized by persistent and not fully reversible airflow limitation, with high economic and social burden, high morbidity and mortality worldwide [1–4]. Recently, Pre-COPD highlighted by the Global Initiative for Chronic Obstructive Lung Disease (GOLD) strategy indicated that there are patients with normal lung function, but with structural abnormalities such as emphysema [2]. Emphysema is characterized by air trapping and dynamic hyperinflation (DH) during exercise [5]. DH is implicated as a major cause of dyspnea during physical activity in COPD, which might cause limited exercise capacity [6, 7]. Exercise is a cornerstone of pulmonary rehabilitation for patients with COPD [8], however, the presence of DH during exercise makes it difficult for patients to tolerate higher training intensities [9].

Therefore, reversing DH is important for patients with COPD or Pre-COPD to reduce dyspnea and improve exercise capacity [10]. Pursed lip breathing (PLB) could theoretically provide positive expiratory pressure (PEP), reduce dynamic compression during expiration, attenuate expiratory airflow restriction, and reduce inspiratory threshold load in hyperinflated lungs in patients with COPD [11]. Thus, PLB has been recommended to reduce dyspnea [12]. However, the increase in airway pressure is due to the increased mouth expiratory resistance, which varies with the rate of airflow, is unstable and cannot be quantified [13]. Therefore, the evidence that PLB is beneficial for dyspnea, exercise endurance, and dynamic hyperinflation remains uncertain. Non-invasive ventilation (NIV) has been used as an aid to improve exercise tolerance in patients with COPD [14]. Among NIV modalities, PEP performs the major role in reversing DH, which can be offered by less costly and elementary devices. The PEP, which is believed to have similarities with PLB, could increase intraluminal airway pressure, counteract intrinsic positive end-expiratory pressure (PEEPi) and reduce airway collapse during expiration by moving the airway equal pressure point from the peripheral airway, which is prone to collapse, to a more solid airway in the center. Thus, PEP could reduce airway collapse and DH, and thus possibly reduce dyspnea and improve exercise capacity [15].

PEP devices have been wildly used to produce a reduction of breathing frequency and expiratory flow limitation, change breathing pattern, improve gas exchange, as well as result in less airway collapse and air-trapping in patients with COPD [16–21]. Russo D et al. had subjects breathe using different levels of PEP (1 cmH_2_O and 10 cmH_2_O) during 6MWT and found that PEP could improve exercise tolerance [18]. Nicolini A et al. had subjects breathe using a simple device with 5 cmH_2_O PEP in 6MWT and found that PEP significantly improved exercise capacity (walking distance), oxygen saturation and heart rate [16]. Cardoso et al. had subjects breathe with PEP device for 20 min and showed that patients had a better physiological breathing pattern with PEP, which was effective in reducing assisted inspiratory muscle activity and improving ventilation and dyspnea [17]. Therefore, previous studies basically used PEP for breathing only during exercise, a brief process.

However, to our knowledge, few studies to date have explored the effects of long-term domiciliary use of PEP devices on exercise capacity in patients with COPD or Pre-COPD. Therefore, this study aimed to evaluate the effects and safety of long-term domiciliary use of PEP delivered by a spring-loaded resistor face mask in patients with COPD or Pre-COPD.

## Methods

### Study subjects

Subjects were consecutive screened from the Pulmonary Clinic of a tertiary care teaching hospital from July 2021 to May 2022. Inclusion criteria: (I) Age range: 18 to 80 years; (II) emphysema on CT scan (HU ≥ − 900); (III) no other imaging changes of lung disease such as occupancy, exudation and interstitial changes on CT scan; (IV) no history of lung cancer, lung resection, cystic fibrosis; (V) signed informed consent. Exclusion criteria: (I) with underlying lung disease such as asthma, bronchiectasis and interstitial lung disease; (II) with heart failure; (III) unwilling to participate. All participants were clinically stable at the time of recruitment. All subjects have not recently, or were currently, participating in pulmonary rehabilitation during the time of the study. Informed consent was obtained from all subjects.

### Study procedures

All enrolled subjects were randomized into intervention group (with PEP device) or control group (with sham-PEP device) by a sequence of computer-generated randomized numbers. An independent third-party researcher was responsible for generating the allocation sequence (i.e., computer-generated random numbers), and the outcome assessors were unaware of patients’ allocation.

**Fig. 1:**
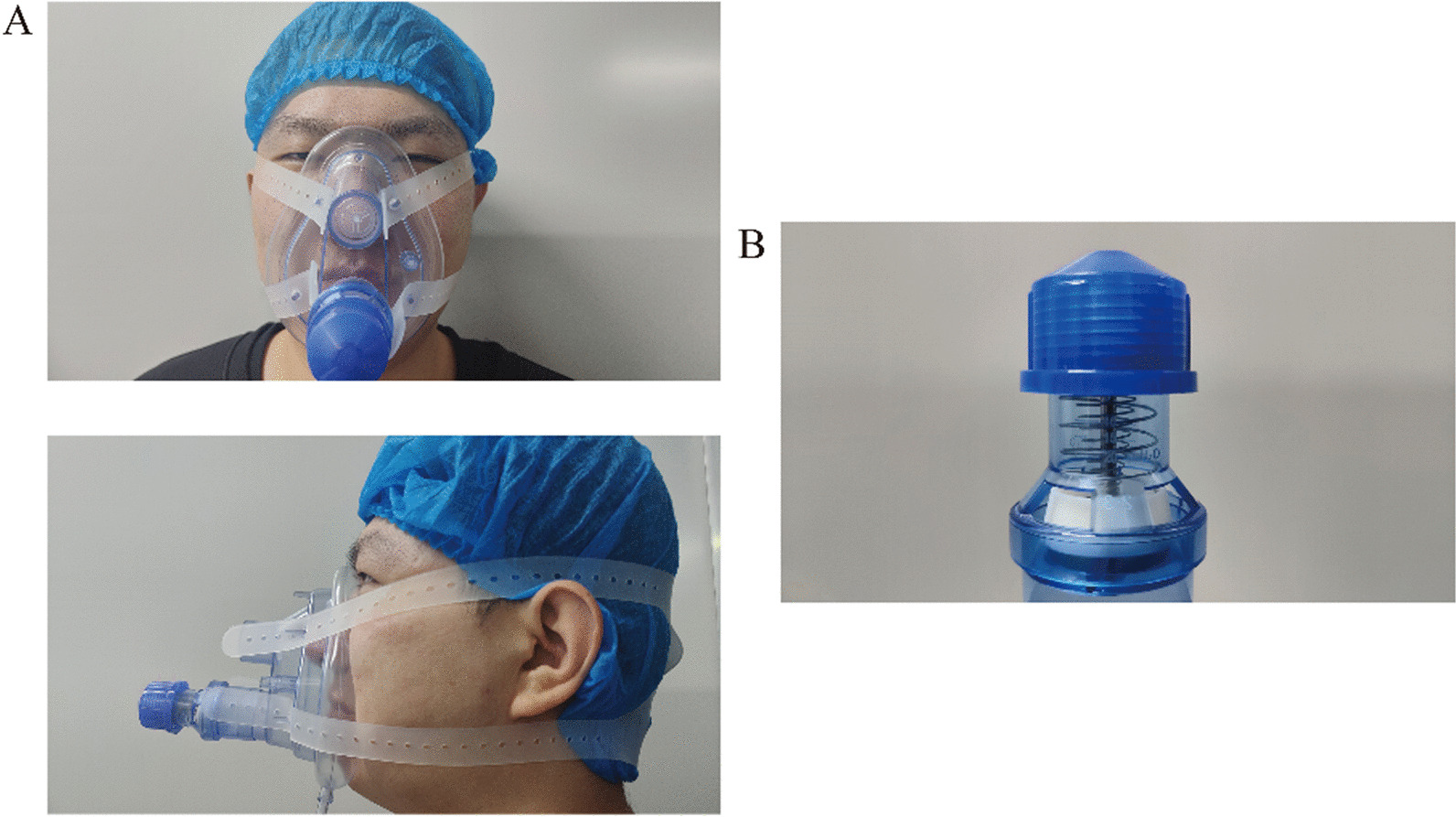
**A** showed a subject in the intervention group using PEP device (PEP = 5cmH_2_O). **B** showed a spring linear pressure resistor. The sham PEP device used in the control group was not loaded with a spring linear pressure resistor

The PEP device was a silicone face mask (PEP device, Xiamen Kangbo Medical facility Company) loaded with a spring linear pressure resistor in a unidirectional expiratory valve and was placed on the subject’s face and held in place with a 4-point strap around the head. The spring linear pressure resistor can be adjustable to provide flow-independent PEP between 5 and 20 cmH_2_O (Fig. 1). During the inspiratory period, the tiny valve opened to lower the inspiratory pressure. In this trial, the resistor was adjusted to perform a load around 5 cmH_2_O and was kept the same for the duration of the study, based on previous studies that used these values and encountered some clinical benefits in patients with COPD during exercise[15, 16]. All subjects needed to do was to put on the mask, inhale through the nose and exhale through the nose. But the subjects needed to make sure that the mask was airtight and did not leak. The sham PEP device was not loaded with a spring linear pressure resistor; thus it did not provide PEP. Subjects were advised to use the PEP device at any time of the day, either continuously or intermittently. Subjects were required to ensure a total of 4 h of use per day for a total of two months and were advised to use the device while sitting or walking slowly, and not during strenuous activities such as running.

During the first visit (T0), the self-administered questionnaire was used to record the demographic and clinical characteristics, including age, weight, gender, medical history, tobacco history, pulmonary function tests, COPD grade, drug treatment, 6-min walk test (6MWT), The partial pressure of end-tidal carbon dioxide (P_ET_CO_2_), and Modified British Medical Research Council score (mMRC score). The breathlessness in daily life was measured by a mMRC score [2]. The P_ET_CO_2_ was measured by portable P_ET_CO_2_ capnography monitor (Kingst®, KMI800). All subjects sat for 10 min to reach a steady state before measuring P_ET_CO_2_, and then were measured P_ET_CO_2_ in a seated position. The pulmonary function was performed (Master Screen Body, Jager, Germany) according to American Thoracic Society/European Respiratory Society (ATS/ERS) guidelines and measured the forced vital capacity (FVC), the forced expiratory volume in one second (FEV1), FEV1/FVC, inspiratory capacity (IC) and maximum voluntary ventilation (MVV) [22]. The 6MWT was performed in a 30-m long, flat, straight, indoor corridor supervised by a well-trained researcher according to the ATS/ERS guidelines [23]. Instructions were provided prior to the 6MWT and all 6MWTs were conducted by the same investigator. The 6-min walk distance (6MWD) was recorded. Heart rate (HR), oxygen saturation (S_P_O_2_) before and after the 6MWT (Initial S_P_O_2_ and End S_P_O_2_) and the minimal S_P_O_2_ during the 6MWT were measured continually through a wrist-worn pulse oximeter (WristOx2, Nonin Medical, Plymouth, MN). Subjects were shown the modified Borg scale before and after the 6MWT to rate their dyspnea and fatigue from 0 to 10, with higher scores indicating worse dyspnea and fatigue. All subjects performed two 6MWTs following the standard procedure mentioned in the guidelines with at least one hour between them and recording the highest 6MWD. After 2 months (T1), during the second visit, the same outcomes were collected and the comfort sensation of using PEP device by a Likert scale from 0 to 10 (0: no discomfort, 10 maximum discomfort) were also collected. For analysis, the ratings were divided into 3 categories: 0 to 3 = ‘not or slightly uncomfortable’, 4 to 6 = ‘moderately uncomfortable’ and 7 to 10 = 'uncomfortable or very uncomfortable’[24]. Primary efficacy outcomes were 6MWD, Borg-Dyspnea and Borg-Fatigue. Primary safety outcomes were P_ET_CO_2_, PEP device-related discomfort and all adverse events. Adverse events associated with the use of the EPAP device were defined as asphyxia, pressure ulcers, acute deterioration, and death. Secondary outcomes were pulmonary function and mMRC score.

Subjects were contacted by phone every 3 days to confirm their adherence to the device. The subjects were asked about their feelings on using the EPAP device and whether they were still using it every day. All subjects were asked this: “Does anything about using this equipment make you uncomfortable?”, “Do you regularly use this device for 4 h every day?”, and “Do you encounter any issues while using this device?” All subjects received optimal medical therapy as usual according to GOLD guidelines at the time of enrollment. Changes to medication were not permitted throughout the study, unless required for safety [2]. If in doubt, subjects were instructed to consult the investigator any time in addition to scheduled phone interviews. All enrolled subjects were explained that two different forms of devices would be compared, but no further details were disclosed, thus, all enrolled subjects were blind on group allocation. All data analyses were performed with the evaluator blinded to the subjects’ condition.

### Statistical analysis

SPSS version 22.0 (SPSS Inc., Chicago, IL, USA) was used for data processing and analysis. Data are presented as mean and standard deviation (mean ± SD), median and interquartile [M(P25, P75)] or frequency counts and percentages. The baseline characteristics were compared using independent samples t-test, Mann–Whitney U test or chi-square test. Paired t-test or Wilcoxon test was used for before-and-after comparisons within the intervention or control groups. Independent sample t test was performed for comparisons between groups. *P* < 0.05 was statistically significant. Subjects were a sample of convenience and the power for this study was 0.82.

## Results

**Fig. 2:**
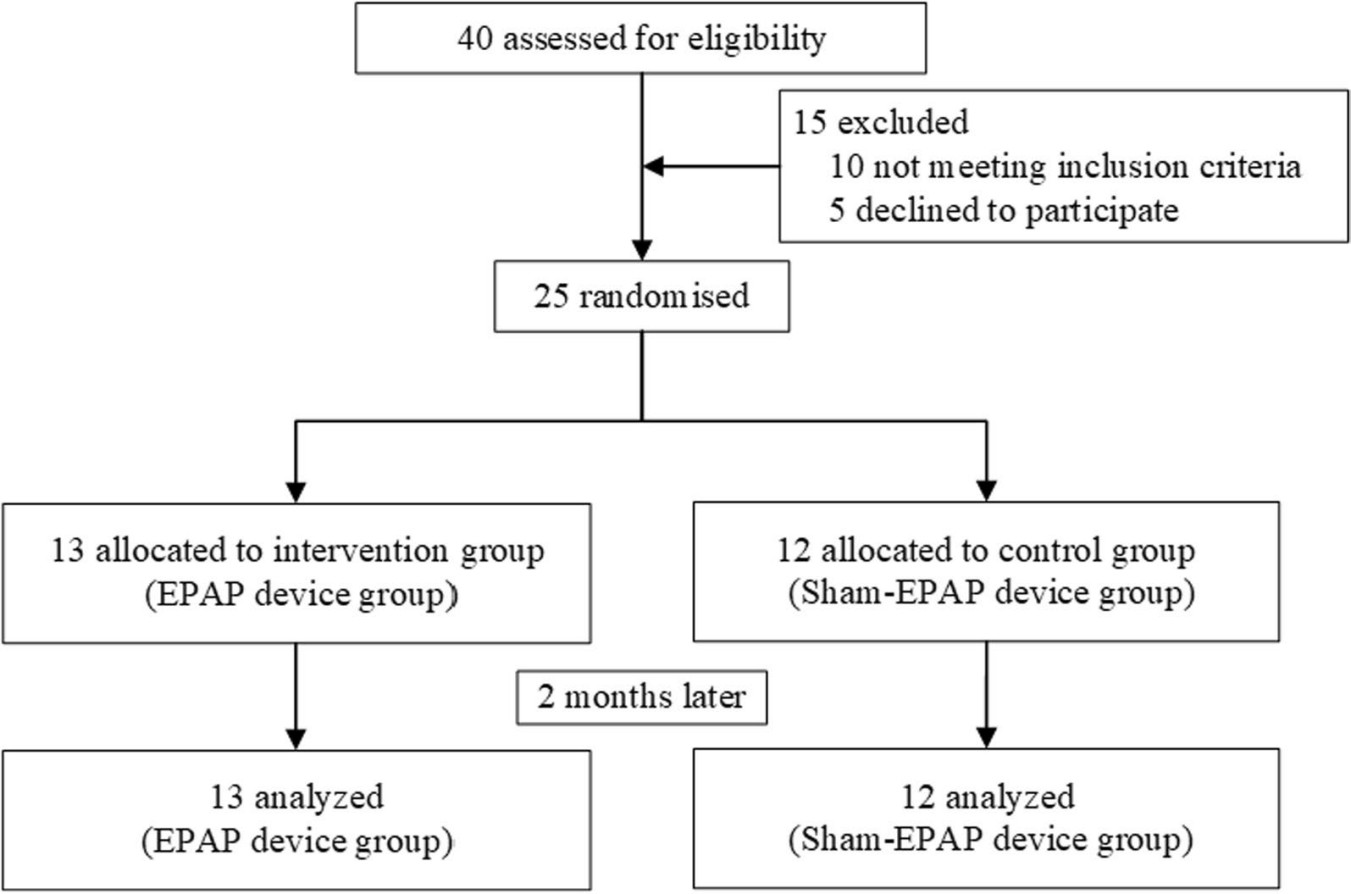
Flow of recruitment of study subjects. A total of 25 subjects were eligible for inclusion and underwent randomization in the study, with 12 subjects in the control group (sham- PEP device) and 13 subjects in the intervention group (PEP device)

Twenty-five consecutive eligible subjects were randomly assigned to either the intervention group (n = 13) or the control group (n = 12), without dropouts and relevant adverse events occurring during the study (Fig. 2). There were no significant differences between two groups at baseline (*P* > 0.05) (Table 1) and no medication changes for all subjects during the whole procedure. Of all subjects who met the inclusion criteria, all current smokers were advised to quit smoking. Until the end of the trial, all subjects were compliant with the training regime.

**Table 1:** Baseline characteristics

baseline characteristics	Intervention group (n = 13)	Control group (n = 12)
Demographic data		
Age, yrs	62 ± 3	54 ± 4
Gender, male/female	12/1	11/1
BMI, kg/m^2^	25 ± 1	24 ± 1
Smoking history, pack-years	39 ± 9	30 ± 7
Pulmonary function tests		
FEV_1_, % predicted	85 ± 6	77 ± 8
FVC, % predicted	102 ± 4	96 ± 8
FEV_1_/ FVC, %	65 ± 4	62 ± 4
IC, % predicted	90 ± 6	96 ± 8
MVV, % predicted	91 ± 7	81 ± 9
COPD grade		
Pre-COPD, %	3 (23.08)	2 (16.67)
GOLD Stage I, %	7 (53.85)	6 (50.00)
GOLD Stage II, %	1 (7.70)	2 (16.67)
GOLD Stage III, %	2 (15.38)	1 (8.33)
GOLD Stage IV, %	0 (0)	1 (8.33)
Treatment		
Inhaled corticosteroids	6 (46.15)	6 (50.00)
Long-acting ß-agonists	8 (61.54)	9 (75.00)
Long-acting muscarinic antagonists	8 (61.54)	10 (83.33)
6MWT		
6MWD, m	545 ± 22	525 ± 31
Initial heart rate, beats/min	82 ± 3	88 ± 4
End heart rate, beats/min	100 ± 6	113 ± 6
Initial S_p_O_2_, %	96 ± 1	96 ± 0
Minimal S_p_O_2_, %	93 ± 1	93 ± 0
End S_p_O_2_, %	95 ± 1	95 ± 0
Initial Borg-Dyspnea	0 (0–0.25)	0 (0–0)
End Borg-Dyspnea	2 (0.75–3)	1 (0.5–2)
Initial Borg-Fatigue	0 (0–0)	0 (0–0)
End Borg-Fatigue	0.5 (0.5–2)	0.75 (0.5–1)
P_ET_CO_2_	33 ± 1	36 ± 1
mMRC score	1 (1–1)	0.5 (0–1.75)

Definition of abbreviations: FEV_1_ = forced expiratory volume in one second; FVC = forced vital capacity; IC = inspiratory capacity; MVV = maximum voluntary ventilation; GOLD = Global Initiative for Obstructive Lung Disease; 6MWD = 6-min walking distance; S_p_O_2_ = oxygen saturation as measured by pulse oximetry; P_ET_CO_2_ = partial pressure of end-tidal carbon dioxide; mMRC score = modified Medical Research Council dyspnea score. Data are shown as n (%), mean ± SD or median ([P25, P75]). All of the baseline characteristics between two groups were similar (all *P* > 0.05)

**Fig. 3:**
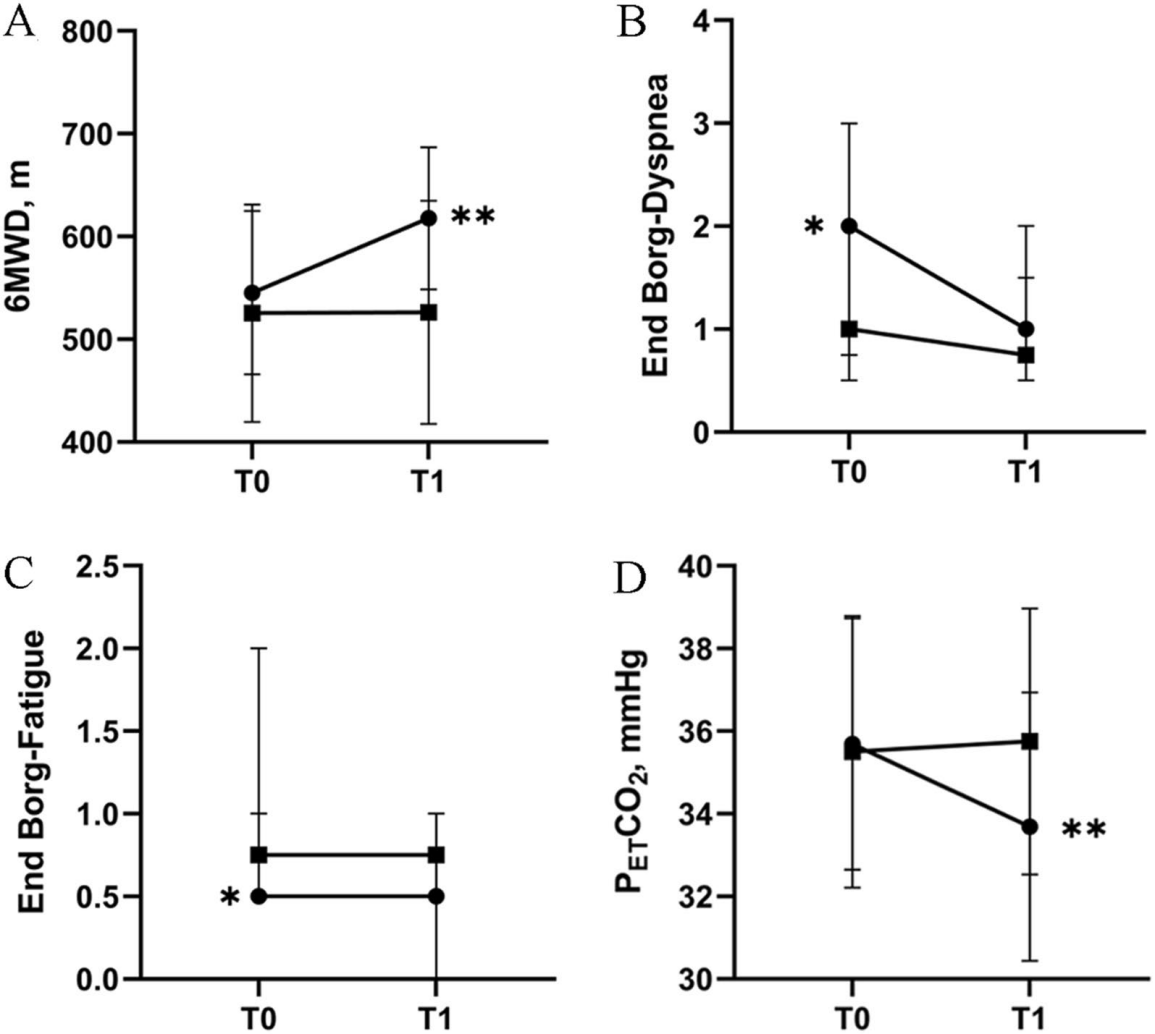
Primary efficacy and safety outcomes. **A** Showed the 6MWD at baseline (T0) and two months later (T1) in the intervention group (solid circles) or control group (solid squares). **B** Showed the Borg-dyspnea score at the end of the 6MWT at baseline (T0) and two months later (T1) in the intervention group (solid circles) or control group (solid squares). **C** Showed the Borg-fatigue score at the end of the 6MWT at baseline (T0) and two months later (T1) in the intervention group (solid circles) or control group (solid squares). **D** Showed the P_ET_CO_2_ at baseline (T0) and two months later (T1) in the intervention group (solid circles) or control group (solid squares). T0 refers to the time when subjects were enrolled. T1 refers to the time when subjects were followed up at 2 months after enrollment. (*: *P* < 0.05; **: *P* < 0.001)

Compared with the control group, 6MWD (− 71.67 ± 8.70 m (95% CI [− 89.67, − 53.67]), *P* < 0.001), end-dyspnea (*P* = 0.002) and end-fatigue (*P* = 0.022) were significantly improved in the intervention group and P_ET_CO_2_ was significantly lower in the intervention group (2.33 ± 0.43 mmHg, (95% CI [1.53, 3.12]), *P* < 0.001). All subjects adopted the PEP device stated that using PEP device in daily life was well acceptable. (Likert scale 1(0.5,1.25)) and there were no complications or adverse events. More details were shown in Table 2 and Fig. 3.

**Table 2:** Primary efficacy and safety outcomes

	Intervention group		*P*	Control group		*P*	Intervention group D (T0 minus T1)	Control group D (T0 minus T1)	*P*
	T0	T1		T0	T1				
6MWD, m	545 ± 79	618 ± 69	< 0.001	525 ± 106	526 ± 109	0.875	− 73 ± 24	− 1 ± 19	< 0.001
Initial Borg-Dyspnea	0 (0–0.25)	0 (0–0)	0.102	0 (0–0)	0 (0–0)	1	0 (0–0.25)	0 (0–0)	0.083
End Borg-Dyspnea	2 (0.75–3)	1 (0.5–1.5)	0.003	1 (0.5–2)	0.75 (0.5–2)	0.257	1 (0.5–1.75)	0 (0–0.38)	0.002
Initial Borg-Fatigue	0 (0–0)	0 (0–0)	1	0 (0–0)	0 (0–0)	1	0 (0–0)	0 (0–0)	1.0
End Borg-Fatigue	0.5 (0.5–2)	0.5 (0–1)	0.016	0.75 (0.5–1)	0.75 (0.5–1)	1	0.5 (0–0.75)	0 (0–0)	0.022
P_ET_CO_2_, mmHg	36 ± 3	34 ± 3	< 0.001	36 ± 3	36 ± 3	0.082	2 ± 1	− 0.25 ± 0.45	< 0.001

Definition of abbreviations: 6MWD = 6-min walking distance; Initial Borg-Dyspnea = Modified Borg scale for dyspnea at the beginning of the 6-min walk test; End Borg-Dyspnea = Modified Borg scale for dyspnea at the end of the 6-min walk test; Initial Borg-Fatigue = Modified Borg scale for fatigue at the beginning of the 6-min walk test; End Borg-Fatigue = Modified Borg scale for fatigue at the end of the 6-min walk test; P_ET_CO_2_ = partial pressure of end-tidal carbon dioxide. Data are shown as mean ± SD or median ([Q1, Q3]). T0 refers to the time when subjects were enrolled. T1 refers to the time when subjects were followed up at 2 months after enrollment. D (Difference) refers to the value at T0 minus the value at T1 (D = T0 minus T1). In order to determine the statistical significance, we used independent sample t test, Mann–Whitney U test, paired t-test or Wilcoxon test

**Fig. 4:**
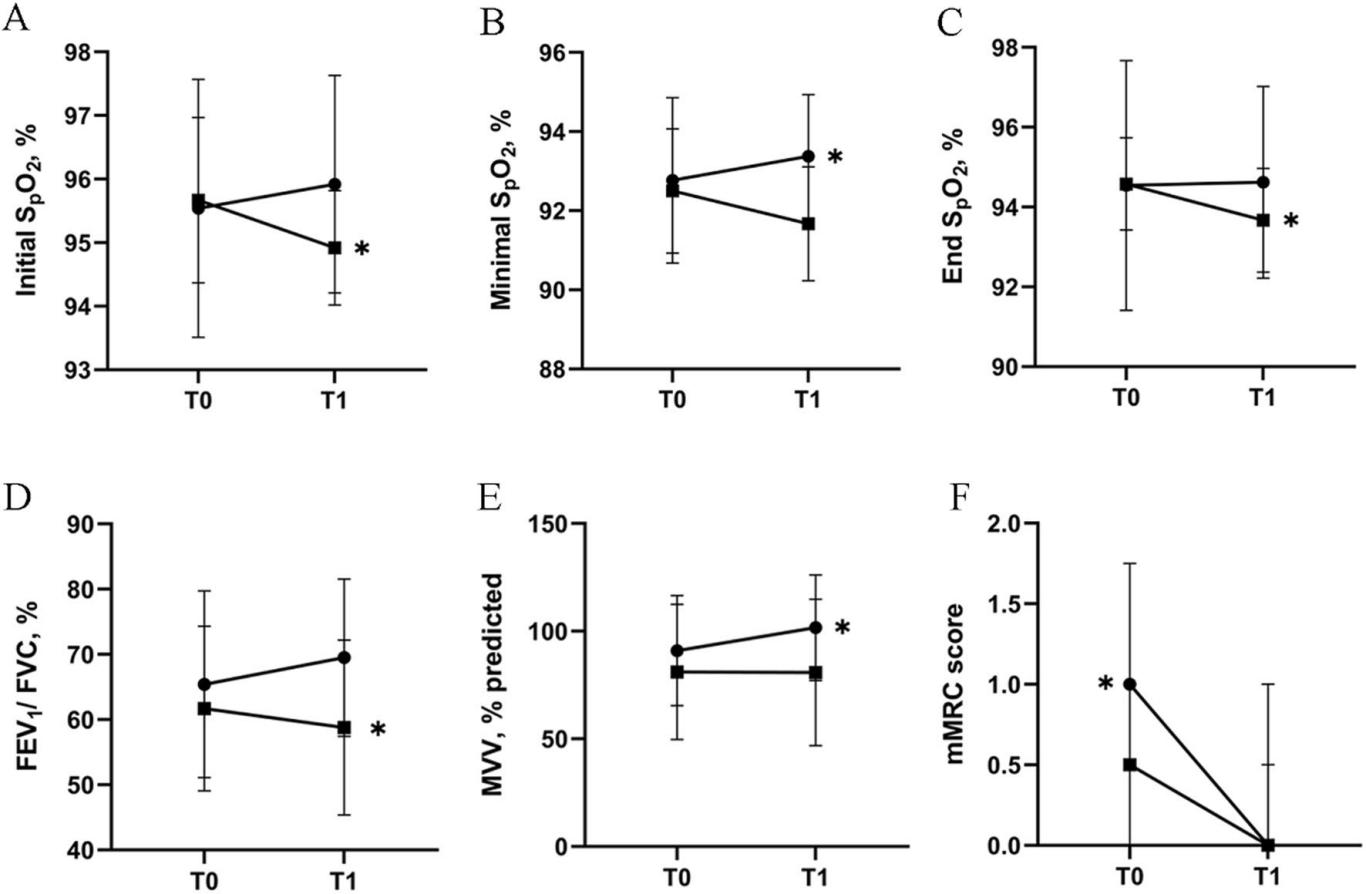
Secondary outcomes. **A** Showed the S_p_O_2_ at the beginning of the 6MWT at baseline (T0) and two months later (T1) in the intervention group (solid circles) or control group (solid squares). **B** Showed the minimal S_p_O_2_ during the 6MWT at baseline (T0) and two months later (T1) in the intervention group (solid circles) or control group (solid squares). **C** Showed the S_p_O_2_ at the end of the 6MWT at baseline (T0) and two months later (T1) in the intervention group (solid circles) or control group (solid squares). **D** Showed the FEV_1_/FVC at baseline (T0) and two months later (T1) in the intervention group (solid circles) or control group (solid squares). **E** Showed the MVV at baseline (T0) and two months later (T1) in the intervention group (solid circles) or control group (solid squares). **F** Showed the mMRC score at baseline (T0) and two months later (T1) in the intervention group (solid circles) or control group (solid squares). T0 refers to the time when subjects were enrolled. T1 refers to the time when subjects were followed up at 2 months after enrollment. (*: *P* < 0.05; **: *P* < 0.001)

Compared with the control group, the initial S_p_O_2_ (1.13 ± 0.39% (95% CI [0.34, 1.93]), *P* = 0.007), minimal S_p_O_2_ (1.45 ± 0.36% (95% CI [0.70, 2.20]), *P* = 0.001), FEV_1_/ FVC (7.00 ± 2.85% (95% CI [1.10, 12.90]), *P* = 0.022) and mMRC (*P* = 0.003) was higher in the intervention group. More details were shown in Table 3 and Fig. 4.

**Table 3:** Secondary outcomes

	Intervention group		*P*	Control group		*P*	Intervention group D (T0-T1)	Control group D (T0-T1)	*P*
	T0	T1		T0	T1				
Initial S_p_O_2_, %	96 ± 2	96 ± 2	0.137	96 ± 1	95 ± 1	0.032	− 0.38 ± 0.87	0.75 ± 0.90	0.007
Minimal S_p_O_2_, %	93 ± 2	93 ± 2	0.025	93 ± 2	92 ± 1	0.01	− 0.62 ± 0.87	0.83 ± 0.94	0.001
End S_p_O_2_, %	95 ± 3	95 ± 2	0.861	95 ± 1	94 ± 1	0.005	− 0.08 ± 1.55	0.92 ± 0.90	0.065
IC, % predicted	90 ± 22	94 ± 22	0.239	96 ± 26	93 ± 24	0.472	− 4.69 ± 13.64	2.85 ± 13.26	0.175
FVC, % predicted	102 ± 13	103 ± 16	0.522	96 ± 28	98 ± 29	0.483	− 1.76 ± 9.65	− 1.55 ± 7.40	0.952
FEV1, % predicted	85 ± 22	91 ± 19	0.173	77 ± 29	74 ± 30	0.129	− 6.46 ± 16.08	2.86 ± 6.03	0.072
FEV1/ FVC, %	65 ± 14	70 ± 12	0.145	62 ± 13	59 ± 13	0.006	− 4.09 ± 9.45	2.92 ± 2.96	0.022
MVV, % predicted	91 ± 26	102 ± 25	0.024	81 ± 31	81 ± 34	0.935	− 10.65 ± 14.86	0.30 ± 12.38	0.058
mMRC score	1 (1, 1)	0 (0, 0.5)	0.002	0.5 (0, 1.75)	0 (0, 1)	0.157	1 (0.5, 1)	0 (0, 0)	0.003

Definition of abbreviations: Initial S_p_O_2_ = oxygen saturation at the beginning of the 6-min walking test; Minimal S_p_O_2_ = minimal oxygen saturation during the 6-min walking test; End S_p_O_2_ = oxygen saturation at the end of the 6-min walking test; IC = inspiratory capacity; FVC = forced vital capacity; FEV_1_ = forced expiratory volume in one second; MVV = maximum voluntary ventilation; mMRC score = modified Medical Research Council dyspnea score. Data are shown as mean ± SD or median ([Q1, Q3]). T0 refers to the time when subjects were enrolled. T1 refers to the time when subjects were followed up at 2 months after enrollment. D (Difference) refers to the value at T0 minus the value at T1 (D = T0 minus T1). In order to determine the statistical significance, we used independent sample t test, Mann–Whitney U test, paired t-test or Wilcoxon test

## Discussion

To the best of our knowledge, this is the first randomized controlled trial to evaluate the effects of using PEP device during activities of daily living on exercise capacity in patients with COPD or Pre-COPD. The major findings of the present study are as follows: (1) Patients in the intervention group showed significant improvement in exercise tolerance after one month of using the PEP device (5 cmH_2_O), as evidenced by improved walking distance, significant improvement in dyspnea and fatigue after 6MWT and significant improvement in minimal S_P_O_2_ during 6MWT (all *P* < 0.05). Meanwhile, the use of the PEP device resulted in a mean 6MWD change of 71.67 ± 8.70 m and a mean Borg scale change in dyspnea at the end of the exercise of 1 (0.5–1.75) units, which were above the established minimal clinically important difference (MCID) [25, 26]; (2) Patients in the intervention group showed significant improvement in FEV_1_/FVC (*P* < 0.05); (3) There was a significant and clinically relevant decrease in activity-related dyspnea in the intervention group (*P* < 0.05), measured with the mMRC scale.

Dyspnea is a common symptom reported by patients with COPD, despite optimal medical management, the use of supplemental oxygen and PLB [27]. PEP could increase bronchial pressure, which can counteract intrinsic positive end-expiratory pressure and move the equal pressure point back to more central and solid airways, alleviate early dynamic airway compression on expiration and attenuate expiratory airflow limitation, which may result in more homogenous ventilation and improved gas exchange [15, 21, 27]. PEP could reduce the inspiratory threshold load of hyperinflated lungs in COPD during exercise, promoting an enhancement in neuromuscular coupling [28]. PEP could change breathing patterns and prolong expiratory time, and thereby reduce lung volumes and DH [19]. PEP devices we used were lower-cost, which facilitated their use in daily life for patients.

Monteiro et al. indicated that a small improvement in DH observed with PEP might lead to greater exercise endurance and reduced dyspnea [21]. However, they suggested that PEP devices increased expiratory effort, which may increase dyspnea and counterbalance the benefits of DH improvement [21]. We observed an improvement in mMRC score and a significant reduction in end-dyspnea after two months of PEP use, and subjects did not experience an increase in dyspnea. It is possible that PEP devices in our study were used during daily life rather than during strenuous exercise on a treadmill as in Monteiro et al. [21]. Another study conducted by Wibmer et al. noted that nasal PEP device reduced DH during 6MWT, but without an improvement in walking distance, possibly because the reduction of DH resulting from short application of the PEP device may not result in an acute improvement in exercise endurance, as subjects might require time to derive an advantage from their improved ventilatory capacity resulting from the reduction in DH [29]. The subjects in our study used the PEP device for 4 h per day for two months to improve DH, and 6MWD was significantly improved. Moreover, the PEP device used by Wibmer et al. provided the PEP of 10 cmH_2_O, and it has been shown that lower level of PEP may be more beneficial [18, 29]. The PEP used in our study was 5 cmH_2_O, which was similar with the pressure produced by PLB [15]. PEP of 5 cmH_2_O improved the exercise tolerance and did not cause significant discomfort such as dyspnea. Vanderschans GP et al. conducted a randomized cross-over controlled trial of eight male patients, all of whom performed two incremental cycling exercises while breathing with or without a PEP device, respectively [15]. They found that PEP may increase dyspnea, which may be related to the high intensity of exercise in their study. The high intensity exercise would make subjects more likely to exertional breathing and make PEP effects underestimated. In another study, subjects in the intervention group was breathing through a flow-dependent PEP device, which may produce a positive pressure of 4–20 cmH_2_O during leg extension exercise. The results showed that the subjects had improved DH, but did not reach statistical significance [20]. They noted that such leg exercising may involve an element of anaerobic metabolism and consequently they may have underestimated the benefit of PEP during purely aerobic exercise such as walking.

Nicolini et al. indicated that PEP (5 cmH_2_O) improved 6MWD and post-exercise S_p_O_2_, which was similar to our findings, and we also found a significant improvement in minimal S_p_O_2_ during 6MWT [16]. The PEP device could prevent the airway collapse, which results in more homogeneous ventilation. Thus, improved ventilation-perfusion distribution probably results in reduced dead space waste, improved alveolar ventilation, and improved S_p_O_2_ [20]. In our study, we observed an improvement in initial S_p_O_2_, which showed that that gas exchange improved after two months. Exercise intolerance in COPD is also associated with reduced IC and occurs with a neuromuscular “power reserve”, which is associated with resting FEV_1_/FVC [30]. IC, which represents the true operating limits for tidal volume expansion in patients with expiratory flow limitation during exercise, is an important surrogate measurement for COPD [31]. The FEV_1_/FVC, one of the spirometry measurements, was associated with expiratory flow limitation and used to classify the airflow limitation severity of patients with COPD [2]. We found there was a significant improvement in FEV_1_/FVC for subjects with EPAP device after 2 months and an improvement in IC without reaching statistical significance in our study, which may be due to IC at baseline having a higher value. Resting IC as a percentage of the predicted value was above 80% for all subjects in our study, which may be due to the fact that the majority of subjects had pre-COPD or mild COPD [7]. Monteiro et al. demonstrated an increase in IC in patients with moderate to severe COPD treated with EPAP at 5 to 10 cmH_2_O after submaximal treadmill exercise [21].

Regarding the safety of PEP devices, our study showed that daily use of PEP devices did not cause carbon dioxide (CO_2_) retention, but rather reduced the P_ET_CO_2_, suggesting that the EPAP device alleviated airway obstruction and improved ventilation. VanderSchans et al.[15] showed that CO_2_ retention were increased in patients with COPD who breathed through a PEP device at 5 cmH_2_O during exercise. They hypothesized that insufficient positive pressure was generated to reduce airway closure and that using higher positive expiratory pressure would be more effective during exercise. Padkao et al.[20] applied conical-PEP with 13 cmH_2_O for patients, and the P_ET_CO_2_ and S_P_O_2_ was not significantly altered, indicating that conical-PEP device with 13 cmH_2_O allowed appropriate gas exchange. Meanwhile, we observed an improvement in S_P_O_2_ in patients with PEP device. These indicated that PEP device used in our study did not have any adverse effects on CO_2_ retention or S_P_O_2_. In addition, the PEP device used in our study proved to be acceptable to the patients when used in daily life. More than 80% of those eligible patients were willing to try it, and of those who were willing, all found it acceptable. When used daily for 4 h a day, there were no adverse effects.

This study has some important limitations. First, the subject sample in our study was small, including subjects with a wide range of disease severity. It can be argued that the heterogeneity of the subjects could have affected the response to the intervention. Therefore, future studies should focus on a larger sample with a more homogeneous population, such as those with consistent disease severity, to avoid underestimating or overestimating the true effect of PEP devices. Second, the recommendation of 4 h of the use of PEP device has no real scientific background. Subjects may accept and tolerate an average of 4 h of PEP device use per day. However, further studies are needed to decide the duration of optimal use. Third, we do not know whether a longer study duration could have resulted in further improvement in lung function, and we expect future studies to clarify these clinically important questions. Fourth, the PEP device used in this study cannot currently be remotely monitored.

## Conclusions

In conclusion, regular daily use of PEP device is safe and may improve exercise capacity in patients with COPD or pre-COPD. Meanwhile, the PEP device may be used in the future to assist COPD patients with breathing retraining and symptom management due to its efficiency, safety, simplicity and low cost when compared to other devices and approaches (such as NIV).

## Data Availability

Data will be available from the corresponding author on reasonable request.

## Abbreviations

COPD: Chronic obstructive pulmonary disease
DH: Dynamic hyperinflation
PLB: Pursed lip breathing
NIV: Noninvasive ventilation
PEP: Positive expiratory pressure
mMRC score: Modified British Medical Research Council score
P_ET_CO_2_: Partial pressure of end-tidal carbon dioxide
6MWT: 6-Minute walk test
GOLD: Global Initiative for Chronic Obstructive Lung Disease
BMI: Body Mass Index
ATS/ERS: American Thoracic Society/European Respiratory Society
FVC: Forced vital capacity
FEV_1_: Forced expiratory volume in one second
IC: Inspiratory capacity
MVV: Maximum voluntary ventilation
6MWD: 6-Minute walking distance
HR: Heart rate
S_P_O_2_: Oxygen saturation
CO_2_: Carbon dioxide

## References

[1] Maltais F, Decramer M, Casaburi R, Barreiro E, Burelle Y, Debigare R, Dekhuijzen PNR, Franssen F, Gayan-Ramirez G, Gea J (2014). An Official American Thoracic Society/European Respiratory Society statement: update on limb muscle dysfunction in chronic obstructive pulmonary disease executive summary. Am J Respir Crit Care Med.

[2] Global Strategy for the diagnosis, management and prevention of COPD, Global Initiative for Chronic Obstructive Lung Disease. GOLD 2021.

[3] Wang C, Xu J, Yang L, Xu Y, Zhang X, Bai C, Kang J, Ran P, Shen H, Wen F (2018). Prevalence and risk factors of chronic obstructive pulmonary disease in China (the China Pulmonary Health [CPH] study): a national cross-sectional study. Lancet.

[4] Organisation WH. The top 10 causes of death. Available at: https://www.whoint/news-room/fact-sheets/detail/the-top-10-causes-of-death (2020).

[5] Rossi A, Aisanov Z, Avdeev S, Di Maria G, Donner CF, Luis Izquierdo J, Roche N, Similowski T, Watz H, Worth H (2015). Mechanisms, assessment and therapeutic implications of lung hyperinflation in COPD. Respir Med.

[6] Agusti A, Hogg JC (2019). Update on the pathogenesis of chronic obstructive pulmonary disease. N Engl J Med.

[7] O'Donnell DE, Elbehairy AF, Webb KA, Neder JA (2017). Canadian Resp Res N: the link between reduced inspiratory capacity and exercise intolerance in chronic obstructive pulmonary disease. Ann Am Thorac Soc.

[8] Spruit MA, Singh SJ, Garvey C, ZuWallack R, Nici L, Rochester C, Hill K, Holland AE, Lareau SC, Man WDC (2013). An Official American Thoracic Society/European Respiratory Society Statement: Key Concepts and Advances in Pulmonary Rehabilitation. Am J Respir Crit Care Med.

[9] Nici L, Donner C, Wouters E, Zuwallack R, Ambrosino N, Bourbeau J, Carone M, Celli B, Engelen M, Fahy B (2006). American thoracic society/European respiratory society statement on pulmonary rehabilitation. Am J Respir Crit Care Med.

[10] O'Donnell DE, Webb KA (2008). The major limitation to exercise performance in COPD is dynamic hyperinflation. J Appl Physiol.

[11] Fregonezi GAD, Resqueti VR, Rous RG (2004). Pursed lips breathing. Arch Bronconeumol.

[12] Pereira de Araujo CL (2015). Karloh M, dos Reis CM, Palu M, Mayer AF. Pursed-lips breathing reduces dynamic hyperinflation induced by activities of daily living test in patients with chronic obstructive pulmonary disease: a randomized crossover study. J Rehabil Med.

[13] Spahija J, de Marchie M, Grassino A (2005). Effects of imposed pursed-lips breathing on respiratory mechanics and dyspnea at rest and during exercise in COPD. Chest.

[14] Dennis CJ, Menadue C, Schneeberger T, Leitl D, Schoenheit-Kenn U, Hoyos CM, Harmer AR, Barnes DJ, Koczulla AR, Kenn K (2021). Bilevel noninvasive ventilation during exercise reduces dynamic hyperinflation and improves cycle endurance time in severe to very severe COPD. Chest.

[15] Vanderschans GP, Dejong W, Devries G, Kaan WA, Postma DS, Koeter GH, Vandermark TW (1994). Effects of positive expiratory pressure breathing during exercise in patients with COPD. Chest.

[16] Nicolini A, Merliak F, Barlascini C. Use of positive expiratory pressure during six minute walk test: results in patients with moderate to severe chronic obstructive pulmonary disease. Multidiscip Respirat Med. 2013, 8. 10.1186/2049-6958-8-19 PMC3637106 23497658

[17] Cardoso DM, Fregonezi GAF, Jost RT, Gass R, Alberton CL, Albuquerque IM, Paiva DN, Barreto SSM (2016). Acute effects of Expiratory Positive Airway Pressure (EPAP) on different levels in ventilation and electrical activity of sternocleidomastoid and parasternal muscles in Chronic Obstructive Pulmonary Disease (COPD) patients: a randomized controlled trial. Braz J Phys Ther.

[18] Russo D, Simonelli C, Paneroni M, Saleri M, Piroddi IMG, Cardinale F, Vitacca M, Nicolini A (2016). Is there an optimal level of positive expiratory pressure (PEP) to improve walking tolerance in patients with severe COPD?. Arch Bronconeumol.

[19] Gass R, Merola P, Monteiro MB, Cardoso DM, Paiva DN, Teixeira PJZ, Knorst MM, Berton DC (2017). Effects of expiratory positive airway pressure on exercise tolerance, dynamic hyperinflation, and dyspnea in COPD. Respir Care.

[20] Padkao T, Boonsawat W, Jones CU (2010). Conical-PEP is safe, reduces lung hyperinflation and contributes to improved exercise endurance in patients with COPD: a randomised cross-over trial. J Physiother.

[21] Monteiro MB, Berton DC, Fontoura Moreira MA, Menna-Barreto SS, Zimermann Teixeira PJ (2012). Effects of expiratory positive airway pressure on dynamic hyperinflation during exercise in patients with COPD. Respir Care.

[22] Miller MR, Hankinson J, Brusasco V, Burgos F, Casaburi R, Coates A, Crapo R, Enright P, van der Grinten CPM, Gustafsson P (2005). Standardisation of spirometry. Eur Respir J.

[23] Crapo RO, Casaburi R, Coates AL, Enright PL, MacIntyre NR, McKay RT, Johnson D, Wanger JS, Zeballos RJ, Bittner V (2002). ATS statement: guidelines for the six-minute walk test. Am J Respir Crit Care Med.

[24] Dawes J (2008). Do data characteristics change according to the number of scale points used? An experiment using 5-point, 7-point and 10-point scales. Int J Mark Res.

[25] Holland AE, Spruit MA, Troosters T, Puhan MA, Pepin V, Saey D, McCormack MC, Carlin BW, Sciurba FC, Pitta F (2014). An official European Respiratory Society/American Thoracic Society technical standard: field walking tests in chronic respiratory disease. Eur Respir J.

[26] Ries AL (2005). Minimally clinically important difference for the UCSD Shortness of Breath Questionnaire, Borg Scale, and Visual Analog Scale. COPD.

[27] Martin AD, Davenport PW (2011). Extrinsic threshold PEEP reduces post-exercise dyspnea in COPD patients: a placebo-controlled, double-blind cross-over study. Cardiopulm Phys Therapy J.

[28] Ambrosino N, Strambi S (2004). New strategies to improve exercise tolerance in chronic obstructive pulmonary disease. Eur Respir J.

[29] Wibmer T, Ruediger S, Heitner C, Kropf-Sanchen C, Blanta I, Stoiber KM, Rottbauer W, Schumann C (2014). Effects of nasal positive expiratory pressure on dynamic hyperinflation and 6-minute walk test in patients with COPD. Respir Care.

[30] Cao M, Calmelat RA, Kierstead P, Carraro N, Stringer WW, Porszasz J, Casaburi R, Rossiter HB (2022). A randomized, crossover, placebo controlled, double-blind trial of the effects of tiotropium-olodaterol on neuromuscular performance during exercise in COPD. J Appl Physiol.

[31] O'Donnell DE, Guenette JA, Maltais F, Webb KA (2012). Decline of resting inspiratory capacity in COPD the impact on breathing pattern, dyspnea, and ventilatory capacity during exercise. Chest.

